# Smart Drug-Delivery Approaches for Enhanced Management of Comorbid Conditions in Alzheimer’s Disease

**DOI:** 10.3390/life16030510

**Published:** 2026-03-19

**Authors:** Gabriela-Dumitrita Stanciu, Ivona Costachescu, Camelia Dascalu, Bogdan-Ionel Tamba

**Affiliations:** 1 Advanced Research and Development Center for Experimental Medicine “Prof. Ostin C. Mungiu”—CEMEX, Grigore T. Popa University of Medicine and Pharmacy Iasi, 700454 Iasi, Romania; 2 Clinical Pharmacology and Algesiology, Department of Pharmacology, Grigore T. Popa University of Medicine and Pharmacy Iasi, 700454 Iasi, Romania

**Keywords:** Alzheimer’s disease, comorbid conditions, smart drug-delivery approaches, preclinical evidence, clinical translation, regulatory challenges

## Abstract

Alzheimer’s disease (AD) remains a major unmet medical challenge due to its complex pathology, high interpatient heterogeneity and frequent association with systemic comorbidities. Conventional pharmacotherapy is limited by poor blood–brain barrier permeability, off-target effects and reduced efficacy in polymedicated elderly populations. Smart drug-delivery systems (DDS), particularly nanotechnology-based platforms, have emerged as promising strategies to enhance brain targeting, optimize controlled drug release and mitigate systemic toxicity. This review examines recent advances in intelligent DDS for AD, with a focus on nanocarriers designed to modulate amyloid aggregation, neuroinflammation, oxidative stress and cholinergic dysfunction. Special attention is given to the impact of the most common comorbid conditions on DDS pharmacokinetics, safety and clinical performance. Furthermore, the challenges associated with clinical translation, such as long-term safety, immunogenicity, manufacturing scalability and regulatory harmonization, are critically discussed. In this context, versatile controlled release platforms that integrate rational design, predictive modeling and Quality by Design-driven manufacturing are highlighted as key enablers of translational success. By bridging intelligent formulation design with scalable production and regulatory readiness, advanced controlled release systems offer a pathway toward precision and patient-centered therapies. Such platforms hold significant potential to accelerate the safe integration of smart DDS into Alzheimer’s disease management and broader neurotherapeutic applications.

## 1. Introduction

Alzheimer’s disease (AD) and related dementias represent one of the most pressing global health and socioeconomic challenges of the twenty-first century [1]. According to the World Health Organization, more than 55 million individuals are currently living with dementia worldwide, with prevalence expected to rise to over 152 million by 2050 due to aging populations and increased life expectancy [1,2]. This growing burden is accompanied by substantial direct healthcare costs and indirect societal impacts, including long-term caregiving, loss of productivity and profound effects on families and caregivers [2,3].

AD is clinically defined by progressive cognitive decline, memory impairment and behavioral changes, and neuropathologically characterized by extracellular beta-amyloid (Aβ) plaques, intracellular neurofibrillary tangles composed of hyperphosphorylated tau, synaptic loss, neuroinflammation and widespread neuronal dysfunction [4,5]. These pathological processes are intertwined with disturbances in neuronal communication, oxidative stress, mitochondrial dysfunction, and chronic inflammation, collectively contributing to irreversible neurodegeneration.

In addition to the complex central nervous system (CNS) pathology, AD frequently co-exists with systemic comorbid conditions that not only increase the risk for the development of AD but also modulate its progression and clinical presentation. Epidemiological evidence has consistently demonstrated associations between AD and type 2 diabetes mellitus, cardiovascular disease, depression, metabolic syndrome and chronic inflammatory disorders [6,7,8,9,10,11,12,13]. These comorbidities share overlapping mechanistic pathways, including insulin resistance, dysregulated lipid metabolism, endothelial dysfunction and chronic low-grade inflammation, which can exacerbate neurodegenerative processes and further compromise cognitive function [14,15]. Moreover, the presence of comorbidities complicates therapeutic management by altering drug absorption, distribution, metabolism and elimination, increasing the likelihood of polypharmacy, drug–drug interactions and adverse effects. Consequently, comprehensive therapeutic strategies must consider both central neurodegeneration and peripheral dysfunctions to optimize patient outcomes.

A critical barrier in the pharmacological management of AD is the restricted access of therapeutic agents to the brain imposed by the blood–brain barrier (BBB). The highly selective nature of the BBB limits the passive diffusion of most small molecules and effectively excludes large biologics, including many monoclonal antibodies and nucleic acid-based therapeutics [16]. This physiological protection contributes to the disappointing translational outcomes of many candidate drugs, which demonstrate promising results in preclinical models but fail to achieve sufficient brain concentrations in clinical settings. Conventional delivery strategies also suffer from rapid systemic clearance, off-target effects and suboptimal pharmacokinetic profiles, further limiting therapeutic efficacy [17,18].

In this context, smart drug-delivery systems have emerged as a transformative approach in neurotherapeutics. These engineered platforms, often based on nanoscale materials such as polymeric nanoparticles, lipid-based carriers, gold nanoparticles, dendrimers, and superparamagnetic iron oxide nanoparticles, are designed to enhance BBB penetration, improve drug stability, prolong systemic circulation and enable controlled or stimuli-responsive release of therapeutic agents [18,19]. Through surface functionalization with targeting ligands, receptor-mediated transport mechanisms can be exploited to facilitate selective accumulation in the CNS, while stimuli-responsive elements allow drug release to be modulated according to local pathological cues, such as pH gradients, oxidative stress or enzymatic activity [19,20].

Importantly, these systems offer opportunities to tackle the therapeutic challenges imposed by AD comorbidities. By enabling the co-delivery of multiple agents, smart nanocarriers can simultaneously address central neurodegeneration and peripheral pathologies. For example, multifunctional platforms can encapsulate neuroprotective drugs alongside antidiabetic, antihypertensive, antidepressant, or anti-inflammatory agents, providing an integrated therapeutic approach that may improve clinical outcomes while reducing systemic toxicity [20]. “Self-regulated” delivery mechanisms, capable of adjusting drug release kinetics or dosing in response to patient-specific factors such as glucose levels, further exemplify the potential for personalized intervention strategies. Despite encouraging preclinical evidence, the translation of these smart delivery systems from bench to bedside remains challenging. Critical issues include large-scale manufacturing, long-term safety, immunogenicity, regulatory approval and cost-effectiveness. Nonetheless, continued innovation in material science, stimuli-responsive designs, and integration with artificial intelligence and precision medicine holds promise for next-generation therapeutic modalities that simultaneously target central and peripheral aspects of Alzheimer’s disease.

While previous studies have focused primarily on individual nanocarrier platforms or their ability to deliver anti-Alzheimer agents, this narrative review offers a novel perspective by integrating the challenges posed by systemic comorbidities with the design and application of multifunctional smart delivery systems. It highlights co-delivery strategies, self-regulated mechanisms, and emerging AI-assisted and personalized approaches as a comprehensive framework for next-generation interventions.

## 2. Major Comorbid Conditions Associated with Alzheimer’s Disease

**Figure 1:**
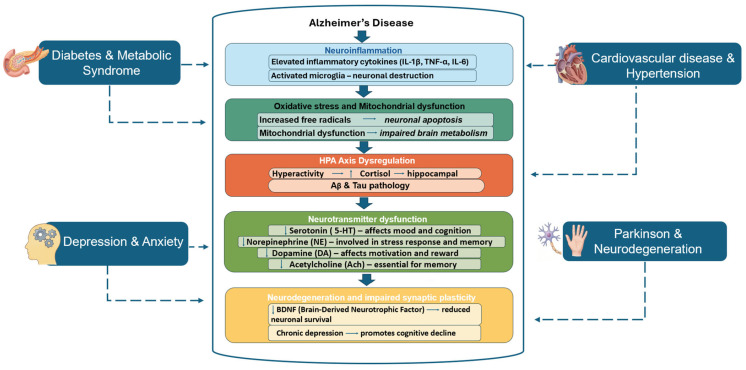
Shared pathophysiological mechanisms linking major comorbidities to Alzheimer’s disease. Age-associated comorbid conditions, including metabolic dysregulation (type 2 diabetes and metabolic syndrome), cardiovascular disease, affective disorders (depression and anxiety), chronic systemic inflammation and parkinsonian syndromes interact with Alzheimer’s disease through overlapping pathophysiological mechanisms. Metabolic disorders contribute to AD pathogenesis primarily through insulin signaling impairment, which disrupts neuronal glucose metabolism and promotes amyloidogenic processing of amyloid precursor protein. Chronic hyperglycemia and insulin resistance further exacerbate oxidative stress and neuroinflammatory responses, facilitating tau hyperphosphorylation and synaptic dysfunction. Vascular dysfunction, endothelial damage and impaired cerebral perfusion can compromise blood–brain barrier integrity and reduce the clearance of amyloid-β peptides. These alterations promote neuroinflammation and accelerate neurodegenerative cascades. Similarly, affective disorders such as depression and anxiety are linked to dysregulation of the HPA axis and chronic elevation of glucocorticoids, which can impair hippocampal neurogenesis, increase oxidative stress and amplify inflammatory signaling pathways involved in AD progression. Parkinsonian syndromes and other neurodegenerative conditions share several pathogenic mechanisms with AD, including mitochondrial dysfunction, impaired proteostasis, defective autophagy and abnormal protein aggregation. These converging mechanisms facilitate the accumulation of misfolded proteins such as Aβ and tau, ultimately contributing to neuronal dysfunction and progressive neurodegeneration. Together, these interconnected pathways highlight the biological links between systemic comorbidities and AD pathology, supporting the concept that Alzheimer’s disease should be considered a multifactorial disorder influenced by both central and peripheral pathological processes. AD, Alzheimer’s disease; HPA axis dysregulation, hypothalamic–pituitary–adrenal; IL-1β, interleukin-1 beta; TNF-a, tumor necrosis factor alpha; IL-6, interleukin-6; Aβ, amyloid-beta; BDNF, brain-derived neurotrophic factor.

Alzheimer’s disease develops in the context of aging-related systemic vulnerability and is frequently accompanied by multiple chronic disorders that shape disease susceptibility, clinical heterogeneity and rates of progression [3]. Accumulating evidence indicates that metabolic, vascular, psychiatric, inflammatory and neurodegenerative comorbidities interact with hallmark AD pathology through overlapping biological pathways that become increasingly dysregulated with age [6,7,8,9,10,11,12,13]. Figure 1 schematically summarizes these interactions, emphasizing convergent mechanisms that link peripheral organ dysfunction to central nervous system neurodegeneration.

### 2.1. Metabolic Dysregulation: Type 2 Diabetes Mellitus and Metabolic Syndrome

Type 2 diabetes mellitus (T2DM) and metabolic syndrome are highly prevalent in aging populations and represent significant risk factors for cognitive decline and dementia, including AD. Multiple epidemiological studies indicate that individuals with T2DM have an elevated risk of developing AD compared with non-diabetic peers, with pooled analyses reporting hazard ratios ranging from approximately 1.5 to 3.0 [21,22].

The proposed mechanistic links between T2DM and AD are thought to begin with neuronal insulin resistance and impaired cerebral glucose metabolism [23]. Insulin plays a crucial role in brain function by modulating synaptic plasticity and supporting neurotrophic signaling. Deficits in insulin signaling have been detected in post-mortem AD brains and are associated with reduced activation of downstream pathways such as PI3K/Akt, which normally inhibit amyloidogenic processes and tau hyperphosphorylation [24].

Preclinical models reinforce this connection: rodents with diet-induced obesity or genetic models of diabetes exhibit exacerbated Aβ deposition, increased tau phosphorylation, compromised hippocampal neurogenesis, impaired long-term potentiation, deficits in learning and memory, and reduced functional performance, which may be interpreted as treatment failure or decreased therapeutic efficacy in these contexts. These observations support the concept that metabolic dysregulation can accelerate core neurodegenerative processes that characterize AD [25,26]. Beyond intrinsic pathology, diabetes and sustained hyperglycemia induce structural and functional alterations in the BBB that have direct implications for drug delivery and pharmacokinetics. Chronic high glucose levels promote oxidative stress, inflammation and upregulation of the glycation end products and receptor for AGEs (AGE–RAGE) axis, which disrupt tight junction integrity and increase BBB permeability, altering the transport of endogenous substrates (e.g., glucose, insulin) and xenobiotics across the cerebral endothelium [27,28]. These changes can result in regional BBB dysfunction, including tight junction disruption and dysregulated expression of key transporters such as P-glycoprotein, low-density lipoprotein receptor-related protein 1 (LRP1) and insulin transporters—all of which shape how drugs and pathological peptides traverse the BBB [29,30].

These pathophysiological alterations have been linked clinically and experimentally to altered drug transport dynamics. Certain antidiabetic agents such as glucagon-like peptide 1 (GLP-1) receptor agonists, sodium-glucose cotransporter 2 inhibitors (SGLT-2) and thiazolidinediones are associated with a reduced risk of dementia in individuals with T2DM [31,32]. In addition, specific antidiabetic drugs may exert neuroprotective effects beyond glucose lowering. For example, GLP-1 receptor agonists enhance insulin signaling in the brain, reduce Aβ deposition and improve synaptic plasticity in preclinical models [33]; SGLT-2 inhibitors may mitigate cerebrovascular dysfunction through improved endothelial function and reduced oxidative stress; and thiazolidinediones activate PPARγ pathways, reducing neuroinflammation and promoting neuronal survival [6,34]. Despite these promising observational findings, results from interventional trials remain mixed. Differences in patient populations, trial duration, cognitive endpoints and drug regimens likely contribute to the variability. Further well-designed, long-term randomized trials are needed to clarify whether these agents can meaningfully prevent or slow cognitive decline in T2DM.

Dyslipidemia and central adiposity, hallmark features of metabolic syndrome, further exacerbate endothelial dysfunction and microvascular injury, leading to widespread compromise of BBB integrity. The resulting BBB disruption impairs cerebral perfusion and reduces the clearance of neurotoxic proteins, including amyloid-β and hyperphosphorylated tau, which accumulate in AD. Mechanistically, dyslipidemia contributes to oxidative stress and inflammation within the cerebrovascular endothelium, while central adiposity promotes chronic low-grade systemic inflammation through the secretion of adipokines and pro-inflammatory cytokines such as tumor necrosis factor alpha (TNF-α) and interleukin-6 (IL-6) [35]. Together, these processes impair cerebrovascular reactivity, disrupt nutrient and oxygen delivery to neurons and facilitate the infiltration of peripheral immune cells into the CNS. Preclinical models have demonstrated that diet-induced obesity or hyperlipidemia leads to exacerbated Aβ deposition, tau pathology and cognitive deficits, providing a mechanistic link between metabolic imbalances and neurodegeneration. Clinically, patients with metabolic syndrome show reduced cerebral perfusion and microvascular rarefaction, correlating with cognitive decline and increased risk of AD [36]. In such patients, systemic metabolic alterations can modify both the pharmacokinetics and CNS availability of neurotherapeutics, leading to either toxicity or suboptimal efficacy. Consideration of metabolic status is therefore critical when evaluating therapeutic outcomes in AD.

### 2.2. Cardiovascular Disease

Cardiovascular disease (CVD) remains a leading cause of morbidity and mortality in aging populations worldwide, and growing evidence implicates atherosclerosis, the central pathogenic substrate of CVD, in the development of late-life cognitive impairment and Alzheimer’s disease [37]. Population-based cohort studies have demonstrated that elevated blood pressure during midlife confers a substantially increased risk of dementia decades later, while cumulative exposure to vascular risk factors across the lifespan correlates with accelerated cortical thinning, hippocampal atrophy and white-matter disruption on neuroimaging. These associations persist even after adjustment for traditional demographic and lifestyle variables, underscore the direct contribution of vascular pathology to neurodegenerative vulnerability [38,39].

At the level of cerebral hemodynamics, vascular aging is accompanied by progressive arterial stiffening, reduced bioavailability of endothelial nitric oxide and impaired neurovascular coupling. Hypertension and atherosclerosis amplify these alterations, compromising autoregulatory capacity and leading to sustained reductions in regional cerebral blood flow [40]. Chronic hypoperfusion preferentially affects watershed territories and metabolically active regions such as the hippocampus and posterior cingulate cortex, where it induces energetic stress, synaptic failure and increased susceptibility to amyloidogenic processing of amyloid precursor protein. In parallel, diminished perivascular drainage and transporter-mediated efflux across the BBB impair Aβ clearance, fostering parenchymal accumulation [41].

Structural and functional disruption of the BBB represents a further link connecting systemic vascular disease to AD pathology. Experimental and human neuropathological studies reveal loss of endothelial tight junction integrity, pericyte degeneration and basement membrane thickening in hypertensive states, changes that have been associated with microglial activation and perivascular inflammation. Such barrier dysfunction facilitates the entry of circulating cytokines, fibrinogen and immune cells into the brain parenchyma, amplifying local inflammatory signaling and oxidative injury to neurons and glia [40,42]. Importantly, these vascular and BBB alterations may also influence the delivery, CNS penetration and pharmacokinetics of neurotherapeutic agents. Impaired transporter activity, disrupted endothelial function and altered cerebral perfusion can reduce drug availability, modify local concentrations, and potentially diminish efficacy or increase toxicity [37,38,39,40,41,42].

Clinico-pathological investigations in aging cohorts consistently indicate that cerebrovascular lesions rarely occur in isolation from classical AD pathology. Lacunar infarcts, cerebral microbleeds, white-matter hyperintensities and arteriolosclerosis frequently coexist with amyloid plaques and neurofibrillary tangles, producing a mixed vascular–Alzheimer’s disease phenotype that dominates in advanced age [43]. This combined pathology is associated with more precipitous cognitive decline, earlier functional dependency and greater neuropsychiatric burden than either disease process alone, highlighting the synergistic rather than additive nature of vascular and neurodegenerative abnormalities [44].

Insights from preclinical models further substantiate these clinical observations. Rodents subjected to chronic hypertension or experimental hypoperfusion develop endothelial dysfunction, breakdown of BBB integrity and exaggerated neuroinflammatory responses. When superimposed on transgenic AD backgrounds, these vascular perturbations accelerate Aβ deposition, intensify tau phosphorylation, and exacerbate synaptic loss and memory impairment [45,46]. Conversely, interventions that improve cerebrovascular function including antihypertensive treatment, restoration of endothelial signaling or enhancement of cerebral perfusion attenuate neuropathological burden and partially rescue cognitive deficits, supporting a causal role for vascular dysfunction in AD pathogenesis [45,46,47].

### 2.3. Psychiatric Comorbidities: Depression and Anxiety in Late Life

Depression and anxiety are among the most common neuropsychiatric comorbidities in Alzheimer’s disease, with prevalence estimates reaching 50% for depressive symptoms and 30% for anxiety in affected cohorts [48,49,50]. Epidemiological studies indicate that late-life anxiety and depression may precede the clinical onset of AD and act as prodromal markers or modifiers of disease progression. A 10-year longitudinal community study reported that clinically significant anxiety at baseline was associated with a nearly threefold increased risk of developing AD, even after adjusting for depression and other confounders [51]. Case–control data from the HUNT study found that depression significantly increased AD risk (OR ~4.39), whereas anxiety showed variable associations depending on dementia subtype [52]. In contrast, the Rotterdam study observed no significant link between anxiety symptoms and dementia risk, highlighting heterogeneity across populations [53]. Cohort data further indicate that individuals with AD have a substantially higher subsequent risk of developing major depression compared with age-matched controls [54].

Preclinical models provide mechanistic support for these clinical observations. APP/PSEN1 transgenic mice display anxiety-like and depression-like behaviors preceding amyloid plaque deposition, mirroring human prodromal features [55]. Similarly, in 3 × Tg-AD mice, pharmacological inhibition of phosphodiesterase-4 (PDE4) with rolipram reduced both neurodegenerative pathology and affective symptoms, implicating neuroinflammation, Aβ accumulation and tau phosphorylation in contributing to the comorbidity of AD and mood dysregulation [56].

Mechanistically, chronic activation of the hypothalamic–pituitary–adrenal (HPA) axis and prolonged glucocorticoid exposure are implicated in both mood dysregulation and AD progression, promoting hippocampal neuronal vulnerability, impairing neurogenesis and enhancing amyloidogenic processing of APP [57]. Elevated systemic inflammatory signaling, evidenced by increased circulating cytokines in depressed patients and stressed animal models, further primes central microglia toward a pro-inflammatory phenotype, exacerbating Aβ and tau pathology [57,58,59]. These mechanistic insights are consistent with findings in APP/PSEN1 and 3 × Tg-AD mice, where HPA axis dysregulation and neuroinflammation accelerate cognitive decline and neuropathological progression [55,56]. These alterations can compromise BBB integrity, modulate drug transporter activity and affect pharmacokinetics, reducing the penetration and efficacy of CNS-targeted therapies. Dysregulated neurochemical signaling associated with affective disorders may further alter receptor responsiveness and pharmacodynamic outcomes [57,58,59].

Together, clinical and preclinical evidence supports a bidirectional and mechanistically convergent relationship between depression/anxiety and AD. Early identification and management of affective symptoms may improve quality of life and modulate neuropathological progression, highlighting the importance of integrated therapeutic strategies for aging populations.

### 2.4. Parkinsonism and Mixed Neurodegenerative Syndromes

Parkinsonian features and mixed neurodegenerative syndromes frequently co-occur with Alzheimer’s disease, contributing to clinical heterogeneity and accelerating cognitive, motor and functional decline. Epidemiological studies indicate that a substantial subset of AD patients present parkinsonian signs, including bradykinesia, rigidity and tremor, often preceding or developing alongside cognitive deficits [60,61]. These motor features are associated with more rapid cognitive deterioration, earlier gait instability, increased neuropsychiatric symptoms and higher rates of falls compared with AD patients without Parkinsonism [62].

Biomarker studies demonstrate that misfolded α-synuclein in cerebrospinal fluid (CSF) often co-exists with phosphorylated tau and elevated Aβ levels, correlating with emergent cognitive deficits in at-risk older adults and suggesting the presence of mixed pathology even in prodromal stages of AD [62,63]. Post-mortem neuropathological analyses reveal overlapping distributions of Aβ, tau and α-synuclein in AD, dementia with Lewy bodies (DLB), and mixed AD/DLB cases, confirming that protein co-pathologies are prevalent in aging brains and contribute to clinical heterogeneity [63,64,65]. Longitudinal imaging studies indicate that Lewy body co-pathology exacerbates regional hypometabolism, synaptic dysfunction and neurodegeneration, accelerating cognitive decline compared with pure AD [66,67].

Preclinical studies provide mechanistic insight into these associations. Transgenic mouse models co-expressing Aβ, tau and α-synuclein recapitulate key features of the human mixed phenotype, including cognitive and motor deficits, synaptic loss, oxidative stress, and enhanced microglial and astrocyte activation [68,69]. These pathological processes may alter transporter activity and receptor responsiveness, potentially reducing the efficacy of CNS-targeted therapies. Pharmacological interventions targeting α-synuclein aggregation, neuroinflammation or mitochondrial dysfunction partially restore cognitive and motor outcomes, demonstrating the translational relevance of these models for testing multi-target therapies [70,71,72].

Together, these findings indicate that Parkinsonism and mixed neurodegenerative syndromes act as accelerators of AD-related neurodegeneration. Recognition of co-pathology is critical for diagnostic accuracy, prognosis and personalized therapeutic strategies, emphasizing the importance of integrated approaches targeting protein aggregation, neuroinflammation and synaptic resilience [62,63,64,65,71,72]. Translationally, preclinical models incorporating both AD and Parkinsonian pathology provide a platform for the development of multi-target therapeutics and biomarker validation, reducing reliance on multiple disease-specific models while supporting ethical and efficient preclinical research.

### 2.5. Chronic Systemic Inflammation and Immune Aging

Chronic systemic inflammation and age-related immune dysregulation (immunosenescence) are increasingly recognized as central contributors to Alzheimer’s disease pathogenesis. Epidemiological studies indicate that elevated peripheral inflammatory markers, including IL-1β, TNF-α, IL-6 and C-reactive protein, predict both incident AD and faster cognitive decline in older adults [73,74]. Longitudinal cohort analyses show that individuals with persistently high systemic inflammation exhibit accelerated hippocampal atrophy, reduced cortical thickness and increased amyloid deposition, supporting a mechanistic link between peripheral immune activation and central neurodegeneration [75,76].

Biomarker studies demonstrate that age-associated immune dysfunction promotes a pro-inflammatory milieu, characterized by dysregulated T cell populations, impaired regulatory networks and chronic low-grade cytokine elevation. This systemic inflammation is mirrored in the central nervous system by microglial priming and astrocytic activation, which exacerbate Aβ accumulation, tau phosphorylation and synaptic dysfunction [77,78]. Chronic cytokine elevation can downregulate hepatic and extrahepatic cytochrome P450 enzymes and drug transporters, altering drug metabolism, clearance and bioavailability. In parallel, inflammation-induced changes in BBB integrity and transporter function can reduce CNS penetration of pharmacological agents, thereby modifying drug efficacy and pharmacodynamic response [79]. Preclinical studies further support a causal role for immune aging in AD. In mouse models, chronic peripheral inflammation accelerates amyloid and tau pathology, induces microglial activation and impairs synaptic plasticity [73,74,80,81]. Conversely, interventions that reduce systemic inflammation, such as anti-cytokine therapies or senolytic agents—ameliorate cognitive deficits and reduce neurodegenerative markers, highlighting translational potential [82,83]. Age-related dysfunction in innate and adaptive immunity also synergizes with other comorbidities, including metabolic syndrome, cardiovascular disease, and neurodegenerative pathologies, amplifying neurodegeneration via convergent inflammatory pathways [35,36,47,54,74].

## 3. Smart Drug-Delivery Systems: Nanotechnology-Based Delivery Approaches

The therapeutic management of Alzheimer’s disease and its prevalent comorbidities remains highly challenging, largely due to limited BBB permeability, rapid systemic clearance, off-target toxicity and subtherapeutic drug concentrations at pathological sites. Smart nanotechnology-based drug delivery systems have emerged as versatile tools capable of enhancing brain targeting, improving pharmacokinetics and enabling temporally and spatially controlled interventions [84]. Such platforms are particularly well-suited to the complex and convergent pathophysiology of AD, which encompasses Aβ and tau aggregation, synaptic dysfunction, neuroinflammation, oxidative stress and mitochondrial impairment. By facilitating precise delivery of small molecules, biologics or nucleic acid therapeutics, nanocarriers can simultaneously modulate multiple pathological pathways, potentially mitigating both core AD pathology and associated comorbid processes such as metabolic dysregulation, vascular dysfunction and mood disorders [85,86].

Preclinical evidence consistently demonstrates that liposomes, polymeric nanoparticles, solid lipid nanoparticles and exosome-like carriers improve central nervous system bioavailability, attenuate neurodegenerative cascades and enhance functional outcomes in AD models [87,88,89,90,91,92,93,94,95,96,97,98,99,100,101,102,103,104,105,106,107,108,109,110,111,112,113,114,115,116,117,118,119,120,121,122,123,124,125,126,127]. Early translational approaches, including intranasal formulations for nose-to-brain delivery and pharmacokinetic optimization studies provide encouraging evidence for clinical applicability and safety profiling. Representative preclinical examples are summarized in Table 1, illustrating the potential of nanotechnology-based systems to address the multifactorial challenges of AD therapy.

Prevalent comorbidities in Alzheimer’s disease critically influence both the pathophysiology and the delivery of therapeutics across the blood–brain barrier. These conditions can impair BBB integrity, exacerbate oxidative stress and alter systemic pharmacokinetics, necessitating design considerations for nanocarrier systems. For instance, neuroinflammation-driven BBB disruption and increased reactive oxygen species highlight the need for multi-mechanistic nanoparticles with anti-inflammatory and antioxidant properties [96,113,120,122,128]. In patients with metabolic comorbidities (e.g., diabetes or hyperlipidemia), biomimetic coatings such as HDL-mimetic, RBC-coated or platelet–CCR2 membrane systems can enhance brain delivery while minimizing off-target effects [95,115,116,117,129,130]. Similarly, vascular comorbidities that reduce perfusion or exacerbate amyloid deposition support the application of nanocarriers exploiting receptor-mediated BBB transport (e.g., B6-SA-SeNPs, sialic acid-modified systems) [120,122,124]. Overall, these considerations demonstrate that smart nanocarrier design must integrate disease- and comorbidity-specific pathophysiological features to optimize targeting efficiency and therapeutic outcomes

## 4. Clinical Translation and Regulatory Challenges

Despite extensive preclinical promise of smart drug-delivery systems (DDS) in overcoming the blood–brain barrier and improving therapeutic outcomes in animal models, no nanoparticle-based therapeutics for Alzheimer’s disease have yet reached advanced clinical trials or regulatory approval. Translation remains limited due to insufficient brain targeting in humans, potential safety and toxicity concerns, complex pharmacokinetics and clearance, and difficulties in scalable manufacturing under Good Manufacturing Practices (GMP) [131,132,133,134,135]. In AD, these challenges are amplified by patient heterogeneity, widespread polypharmacy and disease-associated alterations in BBB permeability [131,136,137,138]. Consequently, biomarker-driven patient stratification and adaptive clinical trial designs are increasingly required to ensure meaningful therapeutic evaluation [137,138,139,140,141]. Bridging this translational gap will require: (i) comprehensive toxicological and pharmacokinetic characterization of nanocarrier physicochemical properties, (ii) robust Quality by Design (QbD)-based scale-up to ensure batch-to-batch consistency, (iii) early engagement with regulatory agencies to establish GMP-compliant manufacturing criteria, and (iv) ethical frameworks that balance technological innovation with patient protection [131,142,143].

The safety profile of smart DDS is of particular importance in AD due to the chronic nature of the disease and the vulnerability of elderly patients with multiple comorbidities. Nanocarriers engineered to cross the altered BBB characterized by increased permeability resulting from amyloid-induced endothelial damage may accumulate in cerebral and peripheral tissues, especially in patients with cardiovascular comorbidities, potentially leading to unintended toxicity [143,144]. Polymeric nanoparticles may persist within the brain, inducing oxidative stress or impairing neuronal function due to incomplete clearance via BBB efflux transporters, the reticuloendothelial system or AD-associated dysfunction of the glymphatic–lymphatic pathways, which exacerbates waste accumulation in aging brains, particularly in individuals with renal insufficiency [131,137,138]. Surface-modified nanocarriers, such as PEGylated liposomes, may also elicit immune responses, including complement activation or cytokine release, potentially aggravating AD-related neuroinflammation and tau pathology [137,145,146]. Importantly, chronic toxicity associated with repeated administration remains insufficiently explored, with limited long-term data on genotoxicity or organ damage, especially in patients with impaired renal function, where reduced clearance may prolong DDS exposure [131,147]. These risks are further compounded by polypharmacy; in polymedicated individuals, drug–drug interactions may compromise safety, as nanocarrier-encapsulated cholinesterase inhibitors can alter the metabolism of concomitant antihypertensive agents, particularly in patients with hepatic insufficiency affecting DDS metabolism [128,134]. Collectively, these gaps highlight the need for chronic toxicity studies in animal models that recapitulate AD with relevant comorbidities, incorporating biomarkers of neurotoxicity and systemic burden. In parallel, post-marketing pharmacovigilance remains essential to capture real-world safety outcomes, including rare hypersensitivity reactions or cumulative toxicity [139].

Scaling smart DDS from laboratory synthesis to industrial production presents significant technological challenges that directly impact clinical translation for AD therapies. Regulatory agencies, including the Food and Drug Administration (FDA), provide specific guidance for liposomal and nanoparticle formulations, covering physicochemical characterization, preclinical safety, pharmacokinetics, biodistribution, and GMP-compliant manufacturing [132,133]. Manufacturing inconsistencies, such as variations in particle size (<100 nm), surface characteristics or drug-loading efficiency, can compromise BBB penetration, release kinetics, and pharmacokinetic predictability, thereby reducing therapeutic efficacy in patients where precise dosing is critical to counteract neuroinflammation and impaired lymphatic clearance [132,134,135]. Formulation stability represents an additional challenge, as nanocarriers may aggregate or degrade during storage, adversely affecting shelf life and therapeutic integrity. This issue is particularly problematic for long-term treatment regimens in patients with renal or hepatic dysfunction, which further influences DDS clearance and metabolism [131,134,135]. Batch-to-batch variability complicates regulatory compliance and quality assurance, underscoring the importance of early process standardization using QbD principles and rigorous preclinical biodistribution and clearance studies, alongside adaptive biomarker-driven clinical monitoring [131,133,142]. Advanced quality control strategies, including in-process monitoring via Process Analytical Technology (PAT), are increasingly recognized as essential to ensure consistent nanomedicine quality [135]. Addressing these manufacturing challenges is critical for the development of scalable, cost-effective DDS aligned with the clinical demands of AD management in comorbid populations [134].

Clinical trial design for smart DDS in AD must rigorously account for patient heterogeneity, disease stage and comorbidities to achieve translational success [137,145]. AD progression is highly variable and influenced by genetic factors, such as APOE genotype, as well as common comorbid conditions (e.g., diabetes and hypertension) that can alter DDS pharmacokinetics via changes in renal clearance or hepatic metabolism [137,145]. The high prevalence of polypharmacy in elderly AD patients further increases the risk of drug–drug interactions, necessitating systematic evaluation of DDS compatibility with concomitant medications [137].

Biomarker-driven patient stratification using amyloid positron emission tomography (PET), cerebrospinal fluid tau measurements or emerging blood-based biomarkers enables targeted enrollment of early-stage patients, who are most likely to benefit from disease-modifying DDS interventions in the context of chronic neuroinflammation [142,145]. Primary endpoints should integrate validated cognitive scales (e.g., ADAS-Cog), functional outcome measures and comorbidity-specific metrics, such as cardiovascular events, with extended follow-up to distinguish sustained clinical benefit from isolated biomarker changes [145]. Unlike conventional pharmacological trials, DDS studies require adaptive designs that allow real-time monitoring of brain penetration and release kinetics through imaging or pharmacodynamic biomarkers, as well as strategies to mitigate attrition associated with cognitive decline [137,139]. Long-term efficacy assessment in comorbid cohorts remains challenging due to confounding factors, emphasizing the need for phase-specific approaches—prioritizing safety and dose optimization in Phase I/II and adequately powered efficacy assessments in later-stage trials [137,145]. Integration of real-world evidence following regulatory approval may further refine stratification strategies and ensure that DDS address the heterogeneous phenotypes encountered in clinical practice [137,145].

The successful clinical translation of DDS for AD ultimately depends on robust ethical and regulatory frameworks that promote innovation while safeguarding vulnerable patients [132,133,142]. Given the frequent impairment of decision-making capacity in AD, informed consent procedures must incorporate legally authorized representatives while preserving patients’ residual autonomy [142]. Long-term safety uncertainties necessitate transparent risk communication and continuous post-marketing surveillance, particularly in elderly individuals with renal or hepatic insufficiency [133,135,142].

Equitable access represents an additional challenge, as the high development and manufacturing costs associated with advanced DDS may limit availability in underserved regions, potentially exacerbating healthcare disparities [133,142]. Regulatory agencies such as the FDA and the European Medicines Agency (EMA) provide guidance for nanomedicines based on case-by-case evaluations of safety and efficacy; however, divergent regulatory requirements exemplified by EMA reflection papers on liposomal formulations versus FDA nanomaterial guidance continue to impede global harmonization and delay widespread clinical adoption [132,133,135]. Furthermore, AD-specific physiological barriers, including BBB dysfunction and compromised glymphatic clearance, must be explicitly addressed within regulatory assessments [137,138]. Ultimately, integrating technological innovation with ethically grounded, patient-centered regulatory strategies and continuous ethical oversight will be essential to ensure that the anticipated benefits of smart DDS outweigh their inherent risks, particularly in comorbid AD populations [133,137,142].

## 5. Future Perspectives

Future advances in Alzheimer’s disease therapy will increasingly depend on the development of smart drug-delivery systems that move beyond disease-centric paradigms toward patient-centered and translationally viable solutions. The marked heterogeneity of AD, compounded by prevalent comorbidities necessitates delivery platforms capable of adapting drug release profiles to individual pathophysiological conditions [6,7,8,9,10,11,12,13]. In this context, versatile controlled release systems that allow modular adjustment of dose, release kinetics and targeting properties represent a critical step toward precision medicine in neurodegenerative disorders.

A key direction for the future is the design of intelligent DDS that respond dynamically to the pathological microenvironment of the AD brain. Stimuli-responsive nanocarriers that sense changes in pH, oxidative stress, enzymatic activity or blood–brain barrier integrity have the potential to enhance therapeutic efficacy while minimizing systemic exposure and cumulative toxicity [15,59,62]. Such adaptive systems are particularly relevant in AD, where BBB permeability and clearance mechanisms evolve throughout disease progression and are further altered by aging and comorbid conditions.

The translation of nanoparticle-based DDS from preclinical models to humans remains challenging. In this context, artificial intelligence provides a complementary dimension by enabling the analysis and interpretation of complex datasets, including neuroimaging, proteomics and longitudinal patient monitoring [148]. Machine learning models have proven useful for identifying early disease signatures, stratifying patients into molecular subtypes, and supporting personalized therapeutic strategies. Concurrently, AI-driven materials informatics can accelerate the optimization of nanoparticle formulations, predict BBB permeability and facilitate adaptive DDS that adjust in real time based on biomarker feedback [149,150].

Integrating AI with predictive pharmacokinetic–pharmacodynamic models enhances the rational design of DDS. Physiologically based pharmacokinetic models combined with AI frameworks allow researchers to simulate nanoparticle transport, brain accumulation and systemic clearance, incorporating patient-specific factors such as genetic background, comorbidities and disease stage [151]. This approach supports individualized dosing and treatment planning while minimizing unnecessary preclinical experiments. The concept of digital twins further enables the iterative refinement of DDS in silico, providing a platform for testing therapeutic strategies under simulated patient-specific conditions [152,153,154].

Equally important is the consideration of scalable and reproducible manufacturing strategies. Many nanotechnology-based DDS fail to progress clinically due to challenges in ensuring batch-to-batch consistency, long-term stability, and regulatory compliance [17,152]. Future platforms should be developed alongside QbD frameworks and real-time process monitoring tools, such as Process Analytical Technology, to maintain robust control over critical material and process attributes. Advanced manufacturing approaches, including microfluidics and additive manufacturing techniques, provide opportunities to produce reliable, clinically relevant formulations [155,156].

Principles derived from versatile controlled release platforms originally developed for advanced topical formulations can be adapted to central nervous system and intranasal delivery strategies. Modular architectures, predictable release kinetics and standardized characterization pipelines enable cross-application of these platforms, facilitating rapid adaptation to diverse therapeutic routes while maintaining consistent performance and safety profiles [157]. This translational flexibility is particularly valuable in neurodegenerative diseases, where alternative routes may help overcome the restrictive properties of the BBB.

Future progress in smart DDS for AD will require early and sustained engagement with regulatory and ethical frameworks. Systems should be designed with regulatory readiness in mind, supported by comprehensive characterization, predictive toxicology and transparent risk–benefit assessment. The growing role of AI in DDS design introduces additional considerations, including data standardization, algorithm transparency and oversight requirements, highlighting the importance of harmonized guidelines to balance innovation with patient safety [158].

By combining AI-assisted design, mechanistic modeling, predictive pharmacokinetics, and scalable manufacturing, versatile controlled-release platforms provide a pathway toward safe, adaptable and clinically translatable therapies for Alzheimer’s disease. Integrating nanotechnology, computational modeling and precision medicine holds the potential to deliver personalized interventions that address the complex, heterogeneous nature of this disorder.

## Figures and Tables

**Table 1:** Nanotechnology-based drug delivery systems in Alzheimer’s disease: Representative preclinical evidence.

Platform	Targeting Strategy/Mechanism	Main Outcomes	Therapeutic Strategy/Advantages	Limitations	Payload/Study Type
**Liposomes**					
Felodipine-modified liposomes	phospholipid bilayers vesicles	modulated endoplasmic reticulum stress, inhibited NLRP3 inflammasome activation, reduced Aβ aggregation and promoted mitophagy, collectively attenuating neuronal apoptosis, improved cognition	- Aβ plaques - high biocompatibility; ability to encapsulate lipophilic drugs; improved drug stability and brain delivery; potential multi-target modulation of AD pathology	possible instability and rapid clearance; limited targeting specificity without ligand functionalization; scalability and long-term safety require further investigation	felodipine (felodipine@LND), 5 × FAD transgenic mice [87]
Transferrin-modified liposomes	receptor-mediated BBB targeting	BBB penetration, decreased neuroinflammation and neuronal apoptosis, enhanced cognitive performance	- neuroinflammation, neuronal apoptosis, BBB dysfunction - efficient receptor-mediated BBB targeting; enhanced brain drug delivery; good biocompatibility	possible off-target uptake in peripheral tissues; stability and large-scale manufacturing challenges	pantothenate (Pan@TRF@Liposome NPs), APPs/PS1 mice [88]
ATX-loaded PEGylation of liposomes	enhanced solubility, BBB permeability	decreased brain endogenous formaldehyde levels, attenuated oxidative stress, reduced Aβ oligomerization and plaque formation, and improved spatial learning and memory	- oxidative stress, Aβ aggregation - improved solubility and stability of hydrophobic drugs; prolonged circulation time; enhanced BBB penetration	potential PEG-related immune responses; limited active targeting without specific ligands; complexity of large-scale production	astaxanthin antioxidant (PEG–ATX@NPs), APPs/PS1 mice [89]
PEG/donepezil liposomes	sustained release and passive BBB penetration	improved brain and plasma bioavailability	- cholinergic dysfunction - sustained drug release; improved pharmacokinetic profile; enhanced brain delivery of donepezil	lack of active targeting; potential PEG-related immune responses; possible drug leakage or stability issues	donepezil, Wistar rats [90] rivastigmine; AlCl_3_-induced AD rats [93]
Imatinib mesylate loaded liposomes	sustained release (up to 96 h); intranasal nose-to-brain delivery enhancing BBB bypass	prolonged drug release; cytotoxic effects up to 25 μg/mL; improved brain penetration and residence time	- Aβ plaques and neuroinflammation - direct nose-to-brain transport bypassing BBB; prolonged drug release; reduced systemic exposure; improved brain bioavailability	variable intranasal absorption; potential mucosal irritation; limited dosing capacity and long-term safety data	imatinib mesylate; in vitro (N2a cells); in vivo (Sprague Dawley rats) [91]
Lecithin and Tween^®^ 80/rivastigmine Liposomes	PEG-DSPE steric hindrance + DDAB electrostatic stabilization; intranasal nose-to-brain delivery	increased rivastigmine’s bioavailability and delayed its release, stable formulation, no tissue toxicity	- cholinergic dysfunction - direct nose-to-brain transport bypassing BBB; improved drug stability and bioavailability; controlled release; reduced systemic exposure	variability of intranasal absorption; potential mucosal irritation; limited drug loading and dosing constraints	rivastigmine; in vivo (rabbits), ex vivo (sheep nasal mucosa) [92]
Soya lecithin/rivastigmine liposomes	intranasal nose-to-brain; liposomal encapsulation	reduced clearance, improved memory in Morris’s water maze and passive avoidance, strong PK-PD correlation with AChE inhibition	- cholinergic dysfunction - direct BBB bypass; enhanced brain bioavailability; protection of drug from rapid metabolism; sustained release	variability in nasal absorption; limited dosing capacity; potential mucosal irritation	rivastigmine; acute scopolamine and chronic colchicine-induced AD rats [94]
**Polymeric nanoparticles**					
Zwitterionic poly(carboxybetaine) (PCB)-based nanoparticle (MCPZFS NP)	BBB penetration; microglia targeting; Aβ recruitment; multi-mechanistic modulation (anti-inflammatory, pro-phagocytic)	reduced proinflammatory cytokines; enhanced Aβ clearance; improved cognition; attenuated Aβ burden; good safety profile	- Aβ plaques and neuroinflammation - enhanced Aβ clearance; anti-inflammatory effects; good biocompatibility and safety profile	complex nanoparticle design and synthesis; limited long-term safety data; translational and large-scale manufacturing challenges	fingolimod + siSTAT3 + ZnO, in vitro (microglia); in vivo (APPs/PS1 mice) [95]
FGL-modified PEG–PTMC(Cit) nanoparticles [FGL-NP(Cit)/HNSS]	BBB and cholinergic neuron targeting; acid-responsive charge switching for lysosomal escape; mitochondrial targeting via SS31 moiety	restored mitochondrial function; reduced Aβ and tau pathology; improved cognition; enhanced antioxidant capacity	- mitochondrial dysfunction, Aβ and tau pathology, oxidative stress - enhanced neuronal and mitochondrial targeting; improved intracellular delivery and lysosomal escape	complex design and synthesis; potential challenges in large-scale production; limited long-term safety and clinical translation data	hybrid peptide HNSS (SS31 + S14G-Humanin), 3 × Tg-AD mice [96]
Oxytocin (OT)-loaded angiopep-2-modified chitosan nanogels (AOC NGs)	LRP1-mediated BBB targeting; microglia modulation	prevented cognitive impairment and delayed hippocampal atrophy	- neuroinflammation and neurodegeneration - enhanced brain delivery; anti-inflammatory and neuroprotective effects; good biocompatibility of chitosan nanogels	possible receptor saturation; limited long-term safety data; stability and large-scale manufacturing challenges	oxytocin, APP/PS1 mice [97]
Multifunctional melanin-like metal ion chelators and neuroinflammation regulators (named PDA@K)	Aβ-binding via KLVFF motif; metal ion chelation; ROS scavenging	reduced Aβ aggregation; decreased oxidative stress	- Aβ aggregation, oxidative stress - potential inhibition of Aβ aggregation	limited in vivo and long-term safety data; unclear pharmacokinetics and BBB transport; translational challenges	melanin-like polydopamine core, in vitro (bEnd.3, BV2, and PC-12 cell lines); in vivo (FAD transgenic mice) [98]
Sugar-based amphiphilic nanoparticles	microglial scavenger receptor targeting	reduced neuroinflammation and Aβ burden	- neuroinflammation and Aβ clearance - specific microglial targeting; enhanced clearance of Aβ; potential anti-inflammatory effects; biocompatible	unclear BBB penetration and pharmacokinetics; challenges in large-scale production	anti-inflammatory agents, BV2 mouse microglia cell line and SH-SY5Y human neuroblastoma cell line [99]
A reactive oxygen species (ROS)-responsive dendrimer-peptide conjugate (APBP)	ROS-triggered release; microglial targeting	reduced ROS level, decreased Aβ burden, alleviated glial cell activation	- neuroinflammation, ROS, and Aβ pathology - multifunctional modulation of oxidative stress and Aβ; good biocompatibility	complex synthesis; limited in vivo and long-term safety data; BBB penetration needs further characterization	peptide therapeutics, APP/PS1 mice [100]
Dual-ligand fusion peptide modified nanoparticles	enhanced BBB penetration; neuron-targeted delivery	improved cognitive function; reduced pathological markers	- AD pathological markers - enhanced BBB penetration; neuron-specific targeting; multi-mechanistic modulation of AD pathology	complex design and synthesis; limited long-term safety data; challenges in large-scale production	neuroprotective agents; in vitro (HT22 cells), in vivo (Aβ-induced mice model) [101]
multifunctional nanoprodrugs (curcumin–hybrid peptide conjugates)	pericyte-targeted delivery; improved BBB penetration	reduced Aβ pathology; improved behavioral performance	- Aβ pathology and neurodegeneration - enhanced BBB penetration; potential neuroprotective effects	complex synthesis; limited in vivo safety and pharmacokinetic data; translational scalability challenges	curcumin conjugates; APP/PS1 mice [102]
Self-destructive nanosweepers	Aβ capture and degradation; enhanced phagocytosis	promoted Aβ clearance; reversed behavioral deficits	- Aβ pathology and neurodegeneration - active Aβ clearance; stimulates microglial phagocytosis; biocompatible	complex design and synthesis; limited in vivo safety and BBB penetration data; scalability challenges	multifunctional peptide–polymer systems; in vitro (N2a cells), in vivo (APP/PS1 mice) [103]
Nanoparticles encapsulating alpha-mangostin	anti-amyloid and antioxidant mechanisms	reduced Aβ aggregation; improved cognition	- Aβ aggregation and ROS - enhanced brain delivery; antioxidant and anti-amyloid effects; improved bioavailability	limited BBB penetration data; in vivo safety and pharmacokinetics not fully characterized; scalability challenges	alpha-mangostin; in vitro (BV-2 cells), in vivo (SAMP8 and SAMR1 mice) [104]
Amorphous PDLLA–dextran bottlebrush copolymers	improved solubility and sustained brain delivery	ameliorated cognitive deficits and oxidative stress	- ROS and cognitive deficits - enhanced solubility and brain delivery; sustained release; biocompatible	limited BBB penetration and pharmacokinetic data; in vivo safety not fully characterized; scalability challenges	hydrophilic antioxidants; in vitro (SH-SY5Y cell), in vivo (SAMP8 mice) [105]
Chitosan/donepezil polymeric	intranasal olfactory delivery for direct nose-to-brain transport	improving donepezil/galantamine/rivastigmine pharmacokinetic characteristics and bioavailability	- cholinergic dysfunction - improved brain bioavailability; enhanced pharmacokinetic profile; biocompatible	variability in nasal absorption; limited dosing capacity; potential mucosal irritation	donepezil; Sprague-Dawley rats [106] galantamine; scopolamine-induced amnesia in Swiss albino mice [107] rivastigmine, Wistar rats [108]
Native poly(D,L-lactide-co-glycolide)-PLGA	direct interaction with hydrophobic domain of Aβ1–42	suppressed spontaneous aggregation of 10 μM Aβ_1–42_ at 25–50 μM PLGA; induced fibril disassembly; reduced tau phosphorylation and ERK1/2 & GSK-3β activation; increased neuronal viability; in 5 × FAD mice: attenuated memory deficits (novel object recognition), reduced cortical Aβ & plaque load; no observable toxicity	- Aβ aggregation, tau pathology - biocompatible and biodegradable; inhibits Aβ aggregation; good in vivo safety profile	limited BBB penetration characterization; pharmacokinetics and long-term safety need further study; scalability challenges	PLGA; in vitro (mouse cortical neurons; iPSC-derived AD neurons); in vivo (5 × FADmice) [109,110,111]
Poly(N-isopropylacrylamide-co-N-tert-butylacrylamide) nanoparticles	bind monomeric and oligomeric Aβ; prolong nucleation lag phase; retard fibrillation kinetics	delayed Aβ fibril formation by extending nucleation phase; inhibited aggregation progression	- Aβ aggregation - inhibits Aβ aggregation; potential disease-modifying effect; biocompatible	limited BBB penetration and pharmacokinetic data; in vivo safety not fully characterized; scalability challenges	native polymeric NPs; in vitro Aβ aggregation assay [112]
PEGylated poly(alkyl cyanoacrylate) nanoparticles	high-affinity binding to Aβ peptides	inhibited Aβ aggregation; decreased Aβ-induced cytotoxicity in neuronal cells	- Aβ aggregation - inhibits aggregation; neuroprotective; PEGylation improves stability and circulation	limited BBB penetration and pharmacokinetic data; in vivo safety not fully characterized; scalability challenges	functionalized with curcumin derivatives or anti-Aβ1–42 antibodies; in vitro neuronal cell models [113]
Iminodiacetic acid-conjugated nanoparticles (IDA-NP)	direct inhibition of Aβ42 fibrillation	reduced metal-induced Aβ aggregation; protected neurons from Aβ cytotoxicity	- Aβ aggregation - direct inhibition of Aβ fibrillation; potential disease-modifying effect	limited BBB penetration and pharmacokinetic data; in vivo safety not fully characterized; scalability challenges	iminodiacetic acid, PC12 cell line [114]
**Biomimetic nanoparticles**					
apolipoprotein E3-reconstituted high-density lipoprotein (ApoE3-rHDL)	biomimetic HDL structure; enhanced BBB crossing; high-affinity binding to Aβ monomers and oligomers; promotion of microglial uptake and lysosomal degradation	reduced Aβ deposition, attenuated microgliosis, ameliorated neurologic changes and rescued memory deficits	- Aβ aggregation and neuroinflammation - promotes Aβ clearance via microglia; multi-mechanistic neuroprotection; biocompatible	complex synthesis; long-term safety and pharmacokinetics need characterization; scalability challenges	ApoE3-functionalized rHDL; in vivo (SAMP8 mice) [115]
Donepezil-loaded ApoA-I rHDL nanoparticles	Aβ clearance via HDL-mimetic binding + AChE inhibition	simultaneous Aβ reduction and cholinesterase inhibition; improved therapeutic efficacy	- Aβ aggregation and cholinergic dysfunction - dual-target therapeutic effect; enhanced BBB penetration; biocompatible	complex synthesis; long-term safety and pharmacokinetics need characterization; scalability challenges	donepezil + ApoA-I rHDL; in vitro (human brain endothelial hCMEC/D3 cells, human SH-SY5Y neuroblastoma cells and murine microglia BV-2 cells) and in vivo (Aβ-induced mouse and rat models) [116]
Cerium oxide nanocrystals in situ on red blood cell membranes (CQD–Ce–RBC)	biomimetic RBC coating for prolonged circulation and biocompatibility	reduced ROS; inhibited Aβ1–42 aggregation; improved cognition; reduced neuroinflammation, TNF-α, IL-1β, IL-6	- ROS, Aβ aggregation and neuroinflammation - prolonged circulation and biocompatibility; multi-mechanistic disease-modifying effect; improved brain delivery	complex synthesis; BBB penetration not fully characterized; scalability challenges	cerium oxide (CeO_2_) nanocrystals + nitrogen-doped carbon quantum dots (CQDs) embedded in red blood cell (RBC) membrane; in vitro (SH-SY5Y neuronal cells), in vivo (APP/PS1 mice) [117]
Hybrid platelet–CCR2 membrane-coated liposomes (TR@CPLs)	enhance BBB penetration and target neuroinflammatory lesions	improved cell viability; significant cognitive improvement; reduced amyloid plaque deposition, glial infiltration and neuroinflammation; no observable systemic toxicity	- neuroinflammation and Aβ pathology - enhanced BBB penetration; neuroprotection; biocompatible; reduced systemic toxicity	complex synthesis; scalability and long-term safety need evaluation; pharmacokinetics in vivo not fully characterized	rapamycin (autophagy enhancer) + TPPU (soluble epoxide hydrolase inhibitor); in vitro (HEK293T cells), in vivo (5xFAD mice) [118]
Biomimetic microglial nanoparticles (MNPs@FMN)	improve BBB penetration and microglial-targeted delivery; FMN-mediated inhibition of riboflavin kinase (RFK) via regulation of KMT2B	ameliorated cognitive deficits, restored synaptic plasticity, reduced hippocampal expression of RFK and pro-inflammatory markers	- neuroinflammation and synaptic dysfunction - enhanced BBB penetration; biomimetic design improves biocompatibility	complex synthesis; long-term safety and pharmacokinetics not fully characterized; scalability challenges	flavin mononucleotide (FMV); in vitro (microglial BV2 cell), in vivo (5 × FAD mice) [119]
- **Inorganic nanoparticles**					
N-acetyl-L-cysteine capped quantum dots (NAC-QDs)	inhibition of Aβ fibrillation	strong inhibition of amyloid fibrillation, suppression of fibril growth and elongation	- Aβ aggregation/fibrillation - potent inhibition of Aβ aggregation; controlled fibril growth	limited BBB penetration data; in vivo safety not fully characterized; potential toxicity of quantum dots; scalability challenges	water-dispersed quantum dots capped with N-acetyl-L-cysteine; in vitro Aβ fibrillation [120]
Gold nanoparticles (AuNPs)	inhibit fibrillization, redirect aggregation toward fragmented fibrils and spherical oligomers	inhibited Aβ fibrillization and reduced neurotoxicity in neuronal cells	- Aβ aggregation - potent Aβ fibrillation inhibition; neuroprotective; facile surface functionalization	limited BBB penetration and in vivo pharmacokinetic data; long-term safety not fully characterized; scalability challenges	bare and carboxyl-conjugated nanoparticles; neuroblastoma cell [121]
Cu2S quantum dots (QDs) functionalized with four cysteine derivatives: N-acetyl-L-cysteine (NAC), N-propionyl-L-cysteine (NPC), N-isobutyryl-L-cysteine (NIBC), and N-pivaloyl-L-cysteine (NPVC)	Aβ_40_ misfolding and fibrillation	suppression of Aβ_40_ aggregation	- direct inhibition of Aβ40 aggregation - potent inhibition of Aβ misfolding and fibrillation; potential imaging functionality	limited BBB penetration and in vivo pharmacokinetic data; potential quantum dot toxicity; long-term safety not fully characterized; scalability challenges	N-acetyl-L-cysteine, N-propionyl-L-cysteine, N-isobutyryl-L-cysteine, N-pivaloyl-L-cysteine; PC-12 cells [122]
Ultra-small C_3_N nanodots	inhibition of Aβ_42_ peptide aggregation	alleviated aggregation-induced cytotoxicity, increasing cell viability; exhibited improved cognitive function	- direct inhibition of Aβ42 aggregation - potent Aβ aggregation inhibition; reduces cytotoxicity; biocompatible	limited BBB penetration and pharmacokinetic data; long-term in vivo safety not fully characterized; scalability challenges	in vitro (primary mouse neurons) and in vivo (APP/PS1 mice) [123]
Sialic acid-modified selenium nanoparticles conjugated with B6 peptide B6-SA-SeNPs)	receptor-mediated endogenous BBB transport systems	enhanced BBB permeability, inhibited Aβ aggregation and protected neuronal cells from Aβ-induced apoptosis	- BBB transport - enhanced BBB penetration; inhibits Aβ aggregation; neuroprotective; biocompatible	limited in vivo pharmacokinetic and long-term safety data; scalability challenges	B6-SA-SeNPs, a synthetic selenoprotein analogue; PC12 and bEnd.3 cells, in vitro BBB Transwell [124]
Chiral L- and D-glutathione-stabilized gold nanoparticles	inhibition activity against Aβ aggregations; BBB permeability	inhibited Aβ_42_ aggregation and crossed the BBB	- BBB transport and inhibition of Aβ aggregation - dual effect: inhibits Aβ aggregation and crosses BBB; biocompatible	limited long-term safety and pharmacokinetic data; scalability challenges	L- and D-glutathione; APP/PS1 mice [125]
Octahedral palladium nanoparticles (Pd NPs) functionalized with polyethylene glycol and borneol (Pd@PEG@Bor)	BBB permeability	reduced intracellular ROS levels, protected mitochondrial integrity, and decreased neuroinflammation, reduced Aβ plaque deposition and improved cognitive function	- BBB penetration - enhanced BBB penetration; neuroprotection; PEGylation improves stability and circulation	limited long-term safety and pharmacokinetic data; potential metal nanoparticle toxicity; scalability challenges	octahedral palladium; in vitro (SH-SY5Y cells) and in vivo (3 × Tg mice) [126]
Ceria/polyoxometalate hybrid nanoparticles (CeONP@POMD)	both proteolytic and superoxide dismutase activities	degraded Aβ monomers and fibrils, inhibited Aβ-induced cytotoxicity, and reduced intracellular ROS; good biocompatibility	- Aβ aggregation and oxidative stress - multi-mechanistic activity; reduces cytotoxicity; good biocompatibility; potential disease-modifying effect	limited BBB penetration and in vivo pharmacokinetic data; long-term safety and scalability not fully characterized	in vitro (PC12 cells and BV2 cells) and in vivo (S4880202 mice) [127]

BBB, blood–brain barrier; ATX-loaded PEGylation of liposomes encapsulating astaxanthin, polyethylene glycol-modified liposomal nanoparticles; NPs, nanoparticles; Aβ, beta-amyloid; ZnO, zinc oxide; siSTAT3, siRNA targeting STAT3; HNSS, mitochondria-targeted hybrid peptide; LRP1, low-density lipoprotein receptor–related protein 1; KLVFF, a self-recognition sequence derived from residues 16–20 of Aβ; ROS, reactive oxygen species; SAMP8 mice, senescence-accelerated prone 8 mice; SAMR1 mice, senescence-accelerated mouse-resistant 1 mice; AlCl_3_, aluminum chloride; PK-PD, pharmacokinetic and pharmacodynamic; PLGA, native poly(D,L-lactide-co-glycolide) nanoparticles; rHDL, reconstituted high-density lipoprotein; apoA-I, apolipoprotein A-I; TPPU, 1-trifluoromethoxyphenyl-3-(1-propionylpiperidin-4-yl) urea; FMV, flavin mononucleotide; RFK, riboflavin kinase; NAC, N-acetyl-L-cysteine; NPC, N-propionyl-L-cysteine; NIBC, N-isobutyryl-L-cysteine; NPVC, N-pivaloyl-L-cysteine.

## Data Availability

All relevant data are included in the article and its references.

## Abbreviations

The following abbreviations are used in this manuscript:

AD	Alzheimer’s disease
Aβ	beta-amyloid
WHO	World Health Organization
BBB	blood–brain barrier
T2DM	type 2 diabetes mellitus
AGEs	advanced glycation end-products
RAGE	receptor for advanced glycation end-products
TNF-α	tumor necrosis factor alpha
IL-6	interleukin-6
GLP-1	glucagon-like peptide 1 receptor agonists
SGLT-2	sodium-glucose cotransporter 2 inhibitors
CNS	central nervous system
CVD	cardiovascular disease
APP/PSEN1 transgenic mice	amyloid precursor protein/presenilin-1 transgenic mice
3 × Tg mice	triple-transgenic Alzheimer’s disease mice (APP Swedish/PSEN1 M146V/Tau P301L)
DLB	dementia with Lewy bodies
CSF	cerebrospinal fluid
ZnO	zinc oxide
siSTAT3	siRNA targeting STAT3
HNSS	mitochondria-targeted hybrid peptide
LRP1	low-density lipoprotein receptor-related protein 1
KLVFF	a self-recognition sequence derived from residues 16–20 of Aβ
ROS	reactive oxygen species
SAMP8 mice	senescence-accelerated prone 8 mice
PLGA nanoparticles	native poly(D,L-lactide-co-glycolide) nanoparticles
SAMR1 mice	senescence-accelerated mouse-resistant 1 mice
rHDL	reconstituted high-density lipoprotein
apoA-I	apolipoprotein A-I
TPPU	1-trifluoromethoxyphenyl-3-(1-propionylpiperidin-4-yl) urea
FMV	flavin mononucleotide
RFK	riboflavin kinase
NAC	N-acetyl-L-cysteine
NPC	N-propionyl-L-cysteine
NIBC	N-isobutyryl-L-cysteine
NPVC	N-pivaloyl-L-cysteine
GMP	good manufacturing practices
DDS	drug-delivery systems
EMA	European Medicines Agency
PAT	process analytical technology
QbD	Quality by Design
FDA	Food and Drug Administration
